# Risk Factors and Management Strategies for Cerebral Amyloid Angiopathy

**DOI:** 10.1093/geroni/igaf122.1010

**Published:** 2025-12-31

**Authors:** Edward Zamrini, Mo-Kyung Sin, Ali Ahmed

**Affiliations:** Irvine Clinical Research, Irvine, California, United States; Seattle University, Seattle, Washington, United States; Washington DC VA Medical Center, Washington, District of Columbia, United States

## Abstract

Cerebral amyloid angiopathy (CAA), amyloid β accumulation in the cerebral blood vessels, is a highly prevalent neuropathology and an independent risk factor for cognitive impairment in older adults. CAA is also a major risk factor for amyloid-related imaging abnormalities (ARIA), the major side effect of anti-amyloid therapies (AATs). As AATs have emerged as a treatment option for Alzheimer’s disease, the importance of CAA is heightened. Better understanding of CAA risk factors without uniquely relying on hemorrhagic imaging markers is of utmost importance to inform potential prevention strategies. Thus, the objectives of this project are 1) to identify patients at high risk for CAA and 2) to provide potential prevention strategies. Most identified CAA risk factors include male gender, old age, smoking, diabetes, heart disease, lower Mini-Mental State Examination scores, APOE 4 carriers, and a deficit in semantic memory. Prevention strategies for risk factor management include lifestyle modification (no smoking, lipid management, exercise to promote cardiometabolic health), and avoiding anticoagulants. While there is no treatment specifically for CAA, any treatment modifying amyloid β accumulation and clearance as well as anti-inflammation could be considered. The need for sensitive and specific non-invasive biomarkers for CAA is also emphasized.

